# Circulating microbiome profiling in transjugular intrahepatic portosystemic shunt patients: 16S rRNA vs. shotgun sequencing

**DOI:** 10.3389/fmed.2025.1662837

**Published:** 2025-12-04

**Authors:** Jingxiang Zhang, Xiaoshuang Xu, Lei Chen, Xincheng Yang, Júlio Ken Matsubara, Yuyao Tian, Jiahua Liu, Xiaoxu Jin, He Chang, Menghui Xu, Chunyang Zhu, Xin Wang, Lingxuan Ren, Jiale Xie, Jiale Liu, Guifen Liu, Mengyao Lu, Xiaowen Wang, Longqi Du, Zilin Ma, Xuechen Liu, Huimin Zhao, Wei Chen, Xiaohui Huo, Guoqi Zheng, Changshun Xie, Chao Xu, Xueqiang Zhang, Wei Qi, Zhijie Feng

**Affiliations:** 1 Hebei Key Laboratory of Gastroenterology, Department of Gastroenterology, Hebei Clinical Research Center for Digestive Diseases, Shijiazhuang, Hebei Institute of Gastroenterology, The Second Hospital of Hebei Medical University, Hebei, China; 2 Marília Medical School, São Paulo, Brazil; 3 Department of Biomedical Engineering, The Chinese University of Hong Kong, Shatin, Hong Kong SAR, China; 4 Department of Neurology, The Second Hospital of Hebei Medical University, China The Key Laboratory of Neurology (Hebei Medical University), Ministry of Education, Shijiazhuang, China; 5 Department of Gastroenterology, Hebei General Hospital, Shijiazhuang, China; 6 Key Laboratory of Tumor Prevention and Precision Diagnosis and Treatment of Hebei, Clinical Oncology Research Center, The Fourth Hospital of Hebei Medical University, Shijiazhuang, China; 7 Department of Gastroenterology, Xingtai People’s Hospital, Xingtai, Hebei, China; 8 Interventional Radiology, Xingtai People’s Hospital, Xingtai, Hebei, China; 9 First Hospital of Hebei Medical University, Shijiazhuang, Hebei, China; 10 Department of Gastroenterology, Cangzhou Central Hospital, Cangzhou, Hebei, China; 11 The First Hospital of Qinhuangdao, Qinhuangdao, Hebei, China; 12 Handan Central Hospital, Handan, Hebei, China

**Keywords:** circulating microbiome, TIPS, shotgun sequencing, 16S rRNA, portal-hepatic circulation

## Abstract

**Background and aim:**

Current efforts to characterize the circulating microbiome are constrained by the lack of standardized protocols for isolating and sequencing microbial communities in blood. To address this challenge, our study compared 16S rRNA (V3-V4 region) and shotgun metagenomic sequencing for circulating microbiome detection.

**Materials and methods:**

After obtaining ethics committee approval and informed consent, samples were aseptically collected from 10 patients undergoing transjugular intrahepatic portosystemic shunt (TIPS) procedures. Shotgun metagenomic reads were taxonomically classified using the Kraken2-Bracken pipeline. 16S rRNA (V3-V4) data were analyzed through an ASV-based approach, with USEARCH for denoising and VSEARCH for taxonomic annotation. The results from both sequencing methods were then systematically compared.

**Results:**

Shotgun metagenomic sequencing generated 7,024,580,376 raw reads (mean depth: 234,152,679.2 reads/sample), while 16S rRNA sequencing produced 6,612,678 raw reads (mean depth: 220,422.6 reads/sample). 16S rRNA amplicon sequencing captured a broader range of microbial signals. Although the taxonomic profiles from both sequencing methods showed limited overlap, the core microbiota common to both were still identified. These conserved core microbial communities exhibited stable α- and β-diversity indices across separate vascular compartments.

**Conclusion:**

In our study, 16S rRNA amplicon sequencing captured more diverse microbial signals than shotgun metagenomics. A stable microbial community structure was observed across vascular compartments, suggesting a homogeneous microbial composition throughout the circulatory system.

## 1 Introduction

Recent studies have revealed extensive alterations in the blood microbiome among liver disease patients. Genera such as *Sphingomonas*, *Bosea*, and *Variovorax* in peripheral blood have been associated with liver fibrosis (1). Additionally, the circulating microbiome of cirrhosis patients showed higher *Enterobacteriaceae* levels and lower abundances of *Akkermansia*, *Rikenellaceae*, and *Erysipelotrichales* (2, 3). Moreover, *Bacteroides* and *Escherichia/Shigella* were significantly more abundant in the hepatic and peripheral veins of patients with severe portal hypertension (2). In patients with hepatocellular carcinoma (HCC), the microbial α-diversity indices in peripheral blood were markedly lower than those observed in cirrhotic individuals or healthy controls (4). The abundance of circulating 16S bacterial DNA rose progressively, increasing from healthy individuals to patients with cirrhosis and escalating further in those with HBV-related acute-on-chronic liver failure (HBV-ACLF) (5).

Due to their low microbial biomass and high host-to-microbial DNA ratio, blood samples pose significant technical challenges for precise microbiome profiling (6–10). Two sequencing approaches are currently utilized. The first method is 16S rRNA gene sequencing, which amplifies hypervariable regions to enable cost-effective taxonomic profiling at the genus level (11–13). The second, whole-genome shotgun metagenomics, requires greater sequencing depth but provides species- and strain-level identification (9, 14–16). However, no study has compared the effectiveness of both methods in characterizing the circulating microbiome.

Moreover, an accurate comparison of these sequencing methods requires both technical optimization and overcoming sample collection challenges. In the transjugular intrahepatic portosystemic shunt (TIPS) procedure, a connection is created between the portal and hepatic veins to alleviate portal hypertension (17, 18). It enables sampling from the portal, hepatic, and peripheral venous systems within the same individual. This established approach not only reduces biological variability between individuals but also creates an ideal setting for systematic methodological comparisons. Critically, portal venous sampling through TIPS provides unparalleled access to the gut-derived microbiome, which is crucial for understanding the pathophysiology of the gut-liver axis (19, 20).

Our study compared the diagnostic potential of 16S rRNA gene sequencing versus shotgun metagenomic sequencing in TIPS-derived whole blood specimens. Although 16S rRNA gene sequencing is more sensitive, the overlap between microbial profiles from both methods may provide stronger evidence for pathophysiological interpretation.

## 2 Materials and methods

### 2.1 Ethics approval

The study protocol was approved by the Ethics Committee of the Second Hospital of Hebei Medical University on March 4, 2022 (approval number: 2022-R177). All participants were provided with a written information sheet and a consent form. The information sheet and the consent form were given for their own review and explained by the research team. Each participant reviewed and signed the consent form to confirm their willingness to participate before enrollment. All procedures were conducted in accordance with the ethical principles of the Declaration of Helsinki (1964) and its later amendments.

### 2.2 Patients recruitment and sample collection

This study was conducted at the Second Hospital of Hebei Medical University. This study enrolled patients aged 18–75 years who were scheduled for their initial elective TIPS. Eligible patients had no significant hepatic encephalopathy, were not pregnant, and provided informed consent. Patients with active severe infections, a history of liver transplantation, or any malignancy were excluded from the study. 10 patients undergoing transjugular intrahepatic portosystemic shunt (TIPS) were recruited between 8 November 2023 and 3 July 2024. During the TIPS procedure, approximately 2 mL of whole blood samples were collected via catheterization using single-use and sterile syringes by experienced interventional radiologists from the portal, hepatic, and peripheral veins immediately prior to shunt placement in the operating suite. The samples were then transferred into EDTA anticoagulant tubes and subsequently transported on ice and promptly stored at −80 °C for preservation.

DNA was isolated from 300 μL of each whole-blood sample using the DNA-Blood Genomic DNA Mini Kit (Aidlab, Cat. No. 20015965) according to the manufacturer’s protocol. Briefly, thawed EDTA-anticoagulated whole blood was first lysed with 900 μL of 1 x red blood cell lysis buffer at room temperature for 10 min to remove erythrocytes. After centrifugation at 12,000 rpm for 20 s, the resulting leukocyte pellet was thoroughly resuspended and treated with 300 μL of nuclear lysis buffer to release genomic DNA. Co-extracted protein were precipitated using 100 μL of protein precipitation solution, followed by centrifugation at 13,000 rpm for 5 min. The supernatant was transferred to a fresh tube and mixed with an equal volume of isopropanol to precipitate DNA. DNA pellets were washed with 70% ethanol, air-dried, and finally dissolved in 100 μL of DNA hydration buffer. DNA concentration and purity were measured using a NanoDrop 2000 spectrophotometer (Thermo), and samples were stored at −80 °C until sequencing (the yield of DNA from each sample is summarized in Supplementary Table 1).

### 2.3 Metagenomic sequencing

DNA libraries for metagenomic sequencing were constructed using 0.02 μg of input DNA with the TruSeq Nano DNA High Throughput Library Prep Kit (Catalog No. 20015965, Illumina) following the manufacturer’s guidelines. The DNA libraries were subsequently sequenced on the Illumina NovaSeq platform using paired-end sequencing with a read length of 2 × 150 base pairs. This workflow generated 7,024,580,376 raw reads, achieving an average sequencing depth of 234,152,679.2 reads per sample.

Quality control of raw sequencing data was carried out using fastp (v0.20.0), which enabled the removal of adapter sequences and trimming of low-quality bases (21). To deplete host-derived sequences, reads were aligned to the GRCh38 human reference genome using BWA (v0.7.18-r1243-dirty) (22). Microbial taxonomic characterization was performed using the Kraken2-Bracken system, in conjunction with the PlusPF database (version: 2024-06-05) which encompasses comprehensive reference genomes spanning archaea, bacteria, viruses, protozoa, and fungi (23, 24).

### 2.4 16 S rRNA sequencing

Targeted amplification of the bacterial 16S rRNA gene V3-V4 hypervariable regions was performed via PCR using specific primer pairs (listed in Supplementary Table 2 for details). Libraries were prepared using the TruSeq Nano DNA LT Library Prep Kit (Illumina, San Diego, CA, United States) according to the manufacturer’s protocol.

The V3-V4 hypervariable regions of the bacterial 16S rRNA gene were amplified using the primer pair (5′-barcode + ACTCCTACGGGAGGCAGCA-3′) and (5′-GGACTACHVGGGTWTCTAAT-3′). Each forward primer contained a unique 7–10 bp barcode sequence to distinguish individual samples within the same library. PCR reactions were performed in a 25μL volume containing 0.25μL DNA polymerase (ABclonal, Wuhan, China), 5 μL of 5 × Reaction Buffer, 5μL of 5 × High GC Buffer, 2 μL of dNTPs (10 mM), 1 μL of forward primer (10 μM), 1 μL of reverse primer (10 μM), 2 μL of template DNA, and 8.75 μL of nuclease-free water. Thermal cycling conditions were as follows: an initial denaturation at 98 °C for 5 min; 25 cycles of 98 °C for 30 s, 52 °C for 30 s, and 72 °C for 45 s; and a final extension at 72 °C for 5 min, with a hold at 12 °C. PCR products were examined on 2% agarose gels, and target bands were excised and purified by a magnetic bead-based cleanup method. Amplicons were quantified using the Quant-iT PicoGreen dsDNA Assay Kit (Invitrogen, P7589) on a BioTek FLx800 microplate reader (recovered concentration in Supplementary Table 3). Equimolar amounts of amplicons were then pooled for library preparation.

The amplified libraries were subsequently subjected to paired-end sequencing with a read length of 2 × 250 base pairs on an Illumina NovaSeq system. This pipeline generated 6,612,678 raw reads, reaching an average sequencing depth of 220,422.6 reads per sample.

To control for potential contamination, UltraPureTM Distilled Water (distilled, deionized, sterile-filtered, and free of DNase, RNase, and protease) was included as a blank control throughout the sequencing process.

Quality control of the raw sequencing data was implemented using VSEARCH (v2.15.2) to filter out low-quality reads (25). Subsequently, dereplication and clustering were performed using USEARCH (v10 and v11), followed by chimera removal (26). This filtering process resulted in 3,060,332 high-quality reads. Amplicon sequence variants (ASVs) were then inferred using VSEARCH. Microbial taxonomic classification was assigned by aligning representative sequences to the Ribosomal Database Project (RDP) 16S rRNA database (v18). A data matrix that includes classifications at various taxonomic levels was generated for downstream analyses.

### 2.5 Taxonomic abundance and richness analyses

For the shotgun metagenomic and 16S rRNA comparison, taxonomic analyses were restricted to bacterial taxa to ensure methodological consistency and comparability between the two sequencing approaches. Archaea, fungi, viruses, and protozoa detected by shotgun sequencing were therefore excluded from the comparative analyses. Relative species abundance (RSA) distributions were calculated for each sample at the genus and phylum levels and visualized using Preston plots. Rarefaction curves were generated with *phyloseq* (1.38.0) and *vegan* (2.6–10) R packages to estimate species richness relative to sequencing depth. RSA skewness was assessed using the *moments* (0.14.1) R package.

### 2.6 Correlation analysis of shared genera between two sequencing methods

Taxonomic annotations from 16S rRNA and shotgun metagenomic data were unified at the genus level using the National Microbial Resource Center database.^1^ Subsequently, genus-level Pearson correlations were used to assess cross-platform agreement in taxonomic abundances, only including genera jointly identified by both 16S rRNA and shotgun metagenomic sequencing.

### 2.7 *In silico* primer-binding analysis

For each genus detected by shotgun metagenomics but not by 16S sequencing, the corresponding reference 16S rRNA gene sequence was obtained from the RDP database (v18). Each reference sequence was individually extracted and used as input for *in silico* amplification with the 338F/806R primer pair. Simulated amplification was performed using *cutadapt* (v4.9) with an error tolerance of 0.1 ( ≤ 10% mismatch), and *seqkit* (v2.3.1) was used for FASTA manipulation and sequence splitting (27, 28). Sequences that failed to produce amplicons under these parameters were classified as non-amplifiable by this primer pair.

### 2.8 Linear regression for method-specific sensitivity

To further characterize method-specific detection sensitivity, linear regression was applied to log-transformed abundances of shared genera. In the primary model, 16S rRNA abundances served as independent variables and shotgun abundances as dependent variables. The reverse model inverted this relationship, using metagenomic data as predictors.

### 2.9 Alpha and beta diversity profiling

Alpha diversity within samples was assessed using Shannon, Simpson, and Chao1 indices, calculated and visualized with the *phyloseq* (1.38.0) and *microbiome* (1.16.0) packages. Group-wise comparisons of alpha diversity were performed using the Wilcoxon Rank-Sum test.

Beta diversity, representing between-sample community dissimilarity, was quantified using Bray-Curtis, Jaccard and Euclidean distances. Clustering patterns were visualized via Principal Coordinate Analysis (PCoA), Non-metric Multidimensional Scaling (NMDS) and Uniform Manifold Approximation and Projection (UMAP). Differences in microbial composition across blood compartments (portal, hepatic, and peripheral veins) were assessed by Permutational Multivariate Analysis of Variance (PERMANOVA). Silhouette analysis was performed to quantify how well samples were separated according to predefined groups in the ordination space.

### 2.10 Statistical analysis

Statistical comparisons followed non-parametric tests (e.g., Wilcoxon rank-sum, PERMANOVA), due to non-normal distributions of microbial abundance data. All statistical analyses and visualizations were performed in R (v4.4.0).

## 3 Results

### 3.1 Demographic and clinical characteristics of the study subjects

The study enrolled 10 cirrhotic patients undergoing TIPS, including 3 females and 7 males, with a mean age of 50.5 ± 17.6 years (Table 1). The underlying etiologies included hepatitis B (*n* = 5), hepatitis C (*n* = 1), fatty liver with chronic cholangitis (*n* = 1), idiopathic cirrhosis (*n* = 1), alcoholic liver disease (*n* = 1), and autoimmune liver disease (*n* = 1). Variceal bleeding was the primary indication for TIPS in 8 patients, while 3 had refractory ascites, and 2 presented with both variceal bleeding and refractory ascites.

**TABLE 1:** Demographic and clinical characteristics of subject groups.

Demographics	ALL (*n* = 10)
Gender (female)	3
Age, mean ± SD	50.5 ± 17.58
**Pathogen**	
Hepatitis B	5
Hepatitis C	1
Fatty liver and chronic cholangitis	1
Idiopathic cirrhosis	1
Alcohol	1
Autoimmune liver disease	1
**Etiology**	
Variceal bleeding	8
Ascites	3
Variceal bleeding + ascites	2
**Biochemical tests**	
Cr, mean ± SD (μmol/L)	59.3 ± 13.6
TBiL, mean ± SD (μmol/L)	26.03 ± 11.52
Na, mean ± SD (mmol/L)	140.14 ± 1.58
INR, mean ± SD	1.26 ± 0.18
**Child-Pugh class**	
Child class A	6
Child class B	3
Child class C	1
Child-Pugh score	6.6 ± 1.85
MELD score	8.3 ± 3.95
MELD-Na score	8.3 ± 3.95
Antibiotics	10
Probiotics	2

Data are displayed as mean ± standard deviation. Cr, creatinine; TBIL, total bilirubin; INR, International Normalized Ratio; MELD score, Model for End Stage Liver Disease.

Disease severity was assessed using the Child-Pugh classification: 6 patients were class A, 3 were class B, and 1 was class C (mean score = 6.6 ± 1.9). The mean MELD score and MELD-Na score were both 8.3 ± 3.9. The mean serum creatinine, total bilirubin, sodium, and INR levels were 59.3 ± 13.6 μmol/L, 260.3 ± 11.5 μmol/L, 140.1 ± 1.6 mmol/L, and 1.26 ± 0.18, respectively. All patients had received antibiotics prior to the procedure, and 2 had concomitant probiotic use.

### 3.2 Comparison of effective reads in 16S rRNA versus shotgun metagenomic sequencing

We performed both 16S rRNA gene and shotgun metagenomic sequencing on the same set of blood samples to evaluate their relative performance. Although both methods were applied to identical material, they yielded substantially different sequencing depths (Table 2). Metagenomic sequencing produced 7,024,580,376 raw reads in total, averaging 234,152,679 ± 37,480,581 reads per sample. Only 722,195 effective reads (24,073 ± 11,517 per sample) were retained following stringent quality control and host DNA removal, yielding 7,192,028 ± 3,422,447 effective base pairs on average.

**TABLE 2:** Summary of raw and effective reads from metagenomic and 16S rRNA sequencing.

	Metagenomic				16S rRNA			
Sample ID	Raw reads	Raw bases (bp)	Effective reads	Effective bases (bp)	Raw reads	Raw bases (bp)	Effective reads	Effective bases (bp)
A_H_000	252,034,480	38,057,206,480	66,387	19,947,338	135,609	66,990,846	125,904	52543197
A_H_001	241,412,394	36,453,271,494	25,086	7,492,868	141,397	69,567,324	131,653	53794370
A_H_003	216,198,192	32,645,926,992	17,861	5,366,558	118,300	58,440,200	109,864	45828113
A_H_005	238,428,056	36,002,636,456	21,667	6,517,829	96,574	47,804,130	89,103	36767440
A_H_006	222,705,564	33,628,540,164	16,598	4,974,954	140,346	69,471,270	126,313	54402756
A_H_007	230,281,628	34,772,525,828	24,311	7,318,810	88,707	43,732,551	82,212	33806112
A_H_008	235,760,932	35,599,900,732	19,604	5,895,587	89,923	44,421,962	83,549	34448829
A_H_009	233,172,968	35,209,118,168	25,207	7,591,220	115,389	57,117,555	106,378	44304860
A_H_010	232,164,274	35,056,805,374	17,710	5,318,969	100,841	49,613,772	92,864	38099748
A_H_011	203,564,898	30,738,299,598	13,534	4,058,100	85,280	42,043,040	79,076	32758094
A_Pe_000	210,842,252	31,837,180,052	30,433	8,504,553	146,549	72,541,755	136,701	56979033
A_Pe_001	232,423,900	35,096,008,900	35,742	10,694,664	142,931	70,464,983	133,796	55810277
A_Pe_003	234,123,962	35,352,718,262	18,766	5,648,828	110,395	54,645,525	102,134	42503184
A_Pe_005	218,214,254	32,950,352,354	20,156	6,060,692	114,156	56,164,752	105,732	43668270
A_Pe_006	228,402,752	34,488,815,552	31,512	9,476,106	146,923	72,286,116	131,581	53644263
A_Pe_007	421,659,336	63,670,559,736	47,050	14,167,415	88,702	43,907,490	82,579	35094263
A_Pe_008	231,115,376	34,898,421,776	20,900	6,289,691	82,305	40,740,975	76,519	31482200
A_Pe_009	233,315,498	35,230,640,198	26,757	8,053,257	82,214	40,449,288	75,908	31410504
A_Pe_010	234,250,710	35,371,857,210	14,760	4,435,178	104,760	51,646,680	97,226	40298388
A_Pe_011	238,560,352	36,022,613,152	15,528	4,656,447	97,261	48,046,934	90,363	38616394
A_Po_000	229,235,564	34,614,570,164	44,045	12,695,508	146,554	72,397,676	136,361	56447863
A_Po_001	245,947,226	37,138,031,126	15,167	4,510,713	137,994	68,307,030	127,570	54806994
A_Po_003	230,431,974	34,795,228,074	19,362	5,818,165	115,420	56,902,060	107,235	44626970
A_Po_005	209,049,128	31,566,418,328	18,705	5,616,913	99,382	49,094,708	92,337	38224014
A_Po_006	215,658,420	32,564,421,420	19,971	5,994,972	131,877	65,147,238	117,524	48909710
A_Po_007	217,209,588	32,798,647,788	23,398	7,039,212	84,865	41,753,580	78,058	32277683
A_Po_008	234,445,008	35,401,196,208	19,785	5,948,122	83,923	41,374,039	77,895	32050198
A_Po_009	237,485,874	35,860,366,974	25,548	7,687,801	105,680	52,205,920	97,750	40515555
A_Po_010	200338468	30,251,108,668	12,041	3,606,600	88,501	43,807,995	81,621	33611935
A_Po_011	216,147,348	32,638,249,548	14,604	4,373,778	91,486	45,011,112	84,526	34881206

Raw Reads: The total number of reads obtained immediately after sequencing, without any quality control or data processing. Effective Reads: The number of high-quality reads retained after filtering for quality and removing host sequences. Raw Bases: The total number of base pairs in the raw sequencing data before any processing. Effective Bases: The total number of high-quality base pairs obtained from the effective reads. bp, base pairs.

In contrast, 16S rRNA sequencing yielded 6,612,678 raw reads in total (220,423 ± 45,760 per sample), and retained a substantially larger number of high-quality microbial reads (3,060,332 total; 102,011 ± 20,918 per sample), corresponding to 42,420,414 ± 8,843,202 effective base pairs per sample.

Notably, despite the substantially higher initial sequencing depth achieved by shotgun metagenomics, the final yield of high-quality microbial reads was considerably lower than that of 16S rRNA sequencing. This highlights the substantial host DNA contamination in whole blood metagenomic libraries and emphasizes the efficiency of 16S rRNA profiling in low-biomass microbiome studies.

### 3.3 Distribution of relative species abundance

**FIGURE 1:**
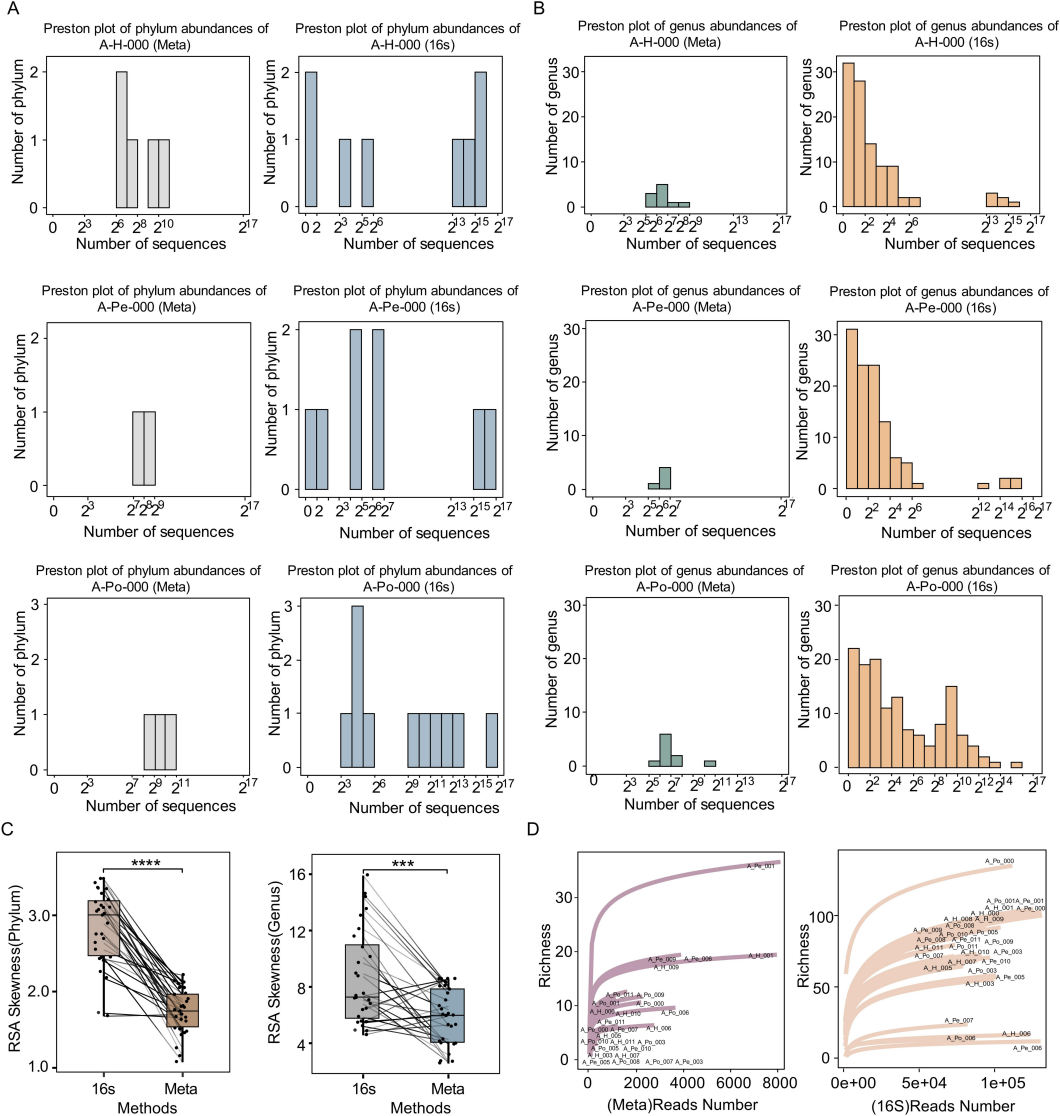
Comparison of microbial community distributions and diversity metrics between 16S rRNA sequencing and shotgun metagenomic sequencing. **(A)** Preston plots showing phylum-level abundance distributions for one representative patient (A-H-000, A-Pe-000, A-Po-000), based on shotgun metagenomic sequencing and 16S rRNA sequencing. **(B)** Preston plots of genus-level abundance distributions for the same samples, derived from shotgun metagenomics and 16S rRNA sequencing. Preston plots are shown on a log2 scale for sequence counts. **(C)** Boxplots of RSA skewness values at the phylum and genus levels, comparing 16S and shotgun sequencing methods. **(D)** Rarefaction curves depicting richness estimates for each sample, sequenced by shotgun metagenomics and 16S rRNA. Sample IDs are labeled. Significance levels: ***P < 0.001, ****P < 0.0001. 16S, 16S rRNA Sequencing; Meta, Shotgun metagenomic sequencing.

In this study, we systematically compared microbial community profiles generated by shotgun metagenomic sequencing and 16S rRNA sequencing, with a particular focus on Relative Species Abundance (RSA) distributions and rarefaction curves. Logarithmically scaled RSA histograms exhibited markedly distinct distributional patterns between the two sequencing methodologies (Figures 1A, B; Supplementary Figures 1–4). The 16S rRNA sequencing displayed a positively skewed RSA distribution. However, the approximate normal distributions were observed in metagenomic sequencing at both phylum and genus levels. Quantitative assessment of distribution skewness further characterized these differences. Although both sequencing strategies produced positively skewed log_2_-transformed RSA distributions, 16S rRNA sequencing exhibited greater skewness than metagenomic sequencing (Figure 1C, paired Student’s *t*-test, *P* = 3.7e-09 and *P* = 0.0022, respectively).

Strikingly, 16S rRNA sequencing identified approximately 10 times more discernible phyla or genera than metagenomic sequencing, indicating its broader taxonomic coverage. Furthermore, 16S rRNA sequencing consistently identified rare phyla and genera within the left-tail of the RSA distribution, a region dominated by low-abundance taxa. This phenomenon may partially explain the positively skewed distribution characteristic of the 16S rRNA sequencing data. Crucially, the rarefaction curves for 16S sequencing also reached a plateau rapidly, demonstrating a relatively lower detection threshold than metagenomics and thereby facilitating the detection of low-abundance taxa (Figure 1D). Collectively, these results established that 16S rRNA sequencing achieves higher sensitivity in profiling microbial taxa.

### 3.4 Genera detection and abundance quantification

**FIGURE 2:**
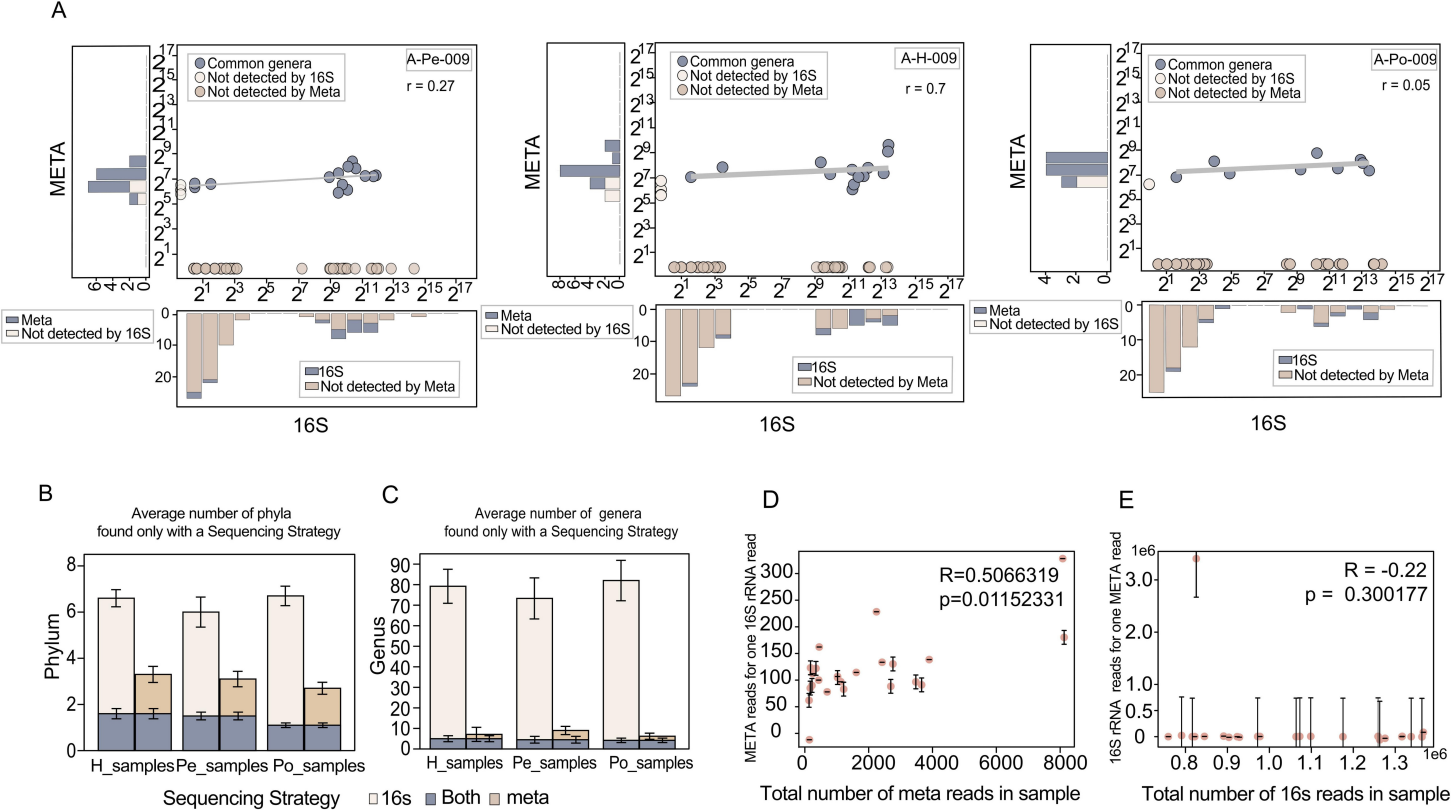
Comparison of microbial taxa detected by 16S and shotgun sequencing across different samples. **(A)** Left and bottom histograms illustrating shared and unique genera detected by each method. Right-side Preston plots showing genus-level abundances from shotgun sequencing in one representative patient (A-H-009, A-Pe-009, A-Po-009). **(B)** Average number of phyla detected exclusively by each sequencing method, both individually and combined, across H, Pe, and Po samples. **(C)** Average number of genera detected exclusively by each sequencing method, both individually and combined, across H, Pe, and Po samples. Error bars in B and C indicate standard error across samples. **(D)** Linear regression intercepts of shotgun versus 16S abundances plotted against the total number of shotgun reads in each dataset, representing the number of shotgun sequences corresponding to one 16S sequence. **(E)** Linear regression intercepts of 16S versus shotgun abundances plotted against the total number of 16S reads in each dataset, representing the number of 16S sequences corresponding to one shotgun sequence. Error bars in D and E indicate the confidence intervals of the intercepts derived from the regression fits. H, hepatic veins blood; Pe, peripheral veins blood; Po, portal veins blood.

To assess the consistency between shotgun metagenomic and 16S rRNA gene sequencing, Pearson correlation coefficients were calculated based on genus shared between the two methods. Overall, the analysis revealed limited concordance between the taxonomic profiles generated by the two approaches. Specifically, the average Pearson correlation coefficients were 0.12 ± 0.22 in the hepatic vein, 0.085 ± 0.20 in the peripheral vein, and 0.172 ± 0.23 in the portal vein (Figure 2A; Supplementary Figure 7).

Notable discrepancies in microbial taxonomic profiles between 16S rRNA and shotgun metagenomic sequencing were consistently observed across hepatic, peripheral, and portal vein samples. Comparative histograms exhibited that most genera identified by shotgun sequencing overlapped with 16S rRNA profiles, whereas a considerable proportion of genera were uniquely captured by the latter (Figure 2A; Supplementary Figures 5, 6). These asymmetrical detection patterns underscored the broader genus-level recovery achieved by 16S rRNA sequencing.

To further assess method-specific differences, we quantified the average number of phyla and genera came from each vascular compartment. At the phylum level, only a small proportion of taxa were shared between 16S rRNA and shotgun metagenomic sequencing, with overlap rates of 16.2% in hepatic vein samples, 16.5% in peripheral ones, and 11.7% in portal ones, respectively (Figure 2B). Genus-level overlap declined markedly, with overlap limited to 5.8% in hepatic vein samples, 5.5% in peripheral ones, and 4.8% portal ones, individually (Figure 2C). These results demonstrated the limited taxonomic concordance between the two sequencing methods.

Given the limited overlap between the two methods, we next examined the taxa detected exclusively by shotgun sequencing using *in silico* amplification analysis with the 338F/806R primer pair. None of these genera showed perfect matches to the primer-binding regions (The 16S-undetected genera and their corresponding reference 16S rRNA sequences are listed in Supplementary Table 5).

Beyond primer mismatch, we observed that these shotgun-only genera occurred sporadically and were confined to a small subset of samples (Supplementary Table 6).

### 3.5 Comparison of detection sensitivity between 16S rRNA and shotgun metagenomics

In the primary regression model, we used 16S rRNA genus-level abundances as the predictors, and noted that the intercept showed a significant positive correlation with total shotgun sequencing depth (*R* = 0.507, *P* = 0.0115) (Figure 2D). This finding suggests that greater sequencing depth improves the sensitivity of shotgun metagenomics in detecting low-abundance taxa.

In contrast, the inverse model revealed no significant correlation between shotgun abundance and 16S sequencing depth (*R* = −0.22, *P* = 0.3002) (Figure 2E), demonstrating that the depths used for 16S rRNA sequencing were sufficient to capture rare microbial signals.

### 3.6 Microbial community structure across vascular compartments

**FIGURE 3:**
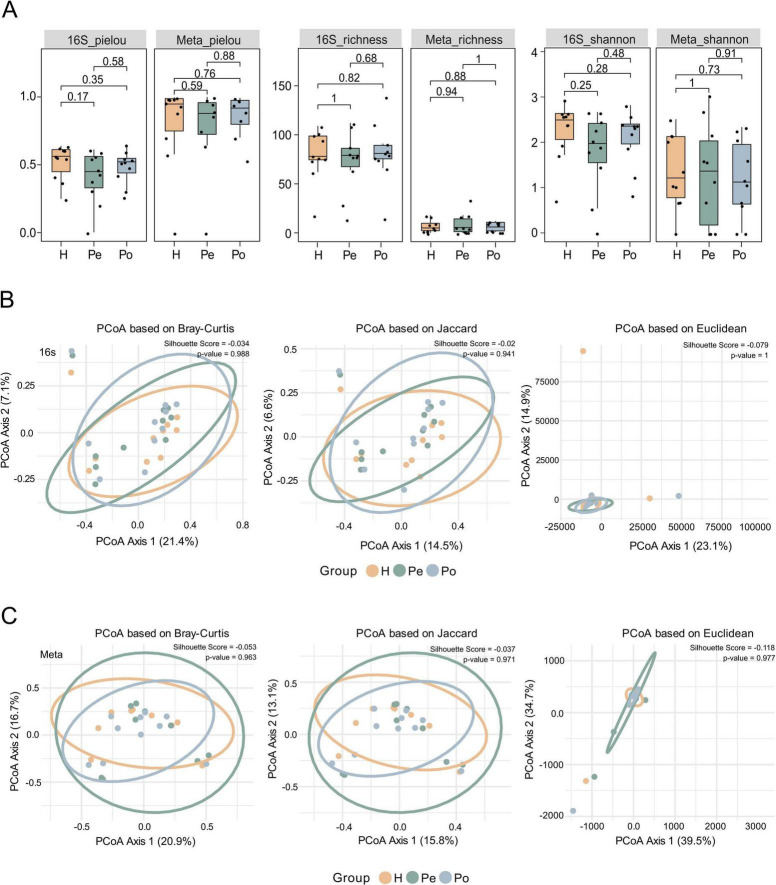
16S rRNA sequencing and metagenomic sequencing show genus-level consistency in stratifying samples across different blood compartments. **(A)** Alpha diversity indices based on Pielou’s evenness, richness and Shannon diversity for 16S sequencing (left) and shotgun metagenomics (right). Statistical significance was evaluated using Wilcoxon rank-sum tests, with p-values annotated. **(B)** Principal coordinate analysis (PCoA) of beta diversity for 16S sequencing based on Bray-Curtis, Jaccard, and Euclidean distance metrics, comparing microbial communities among H (hepatic), Pe (peripheral), and Po (portal) vein samples. **(C)** PCoA of beta diversity for shotgun metagenomics using the same three distance metrics, similarly assessing differences among groups.

Microbial diversity was assessed across hepatic (H), peripheral (Pe), and portal (Po) vein samples at the genus level using both sequencing platforms. with no significant differences in alpha diversity (Figure 3A). Similarly, beta diversity based on Bray-Curtis, Jaccard, and Euclidean distances dissimilarity showed no significant difference between each group (Figures 3B, C; Supplementary Figure 8) (*P* > 0.05). Negative silhouette scores further supported the absence of compartment-specific structure. Consistently, reference-free k-mer—based PCoA analysis using *Sourmash* (4.9.4) also revealed no compartment- or methods-specific clustering (Supplementary Figure 9).

**FIGURE 4:**
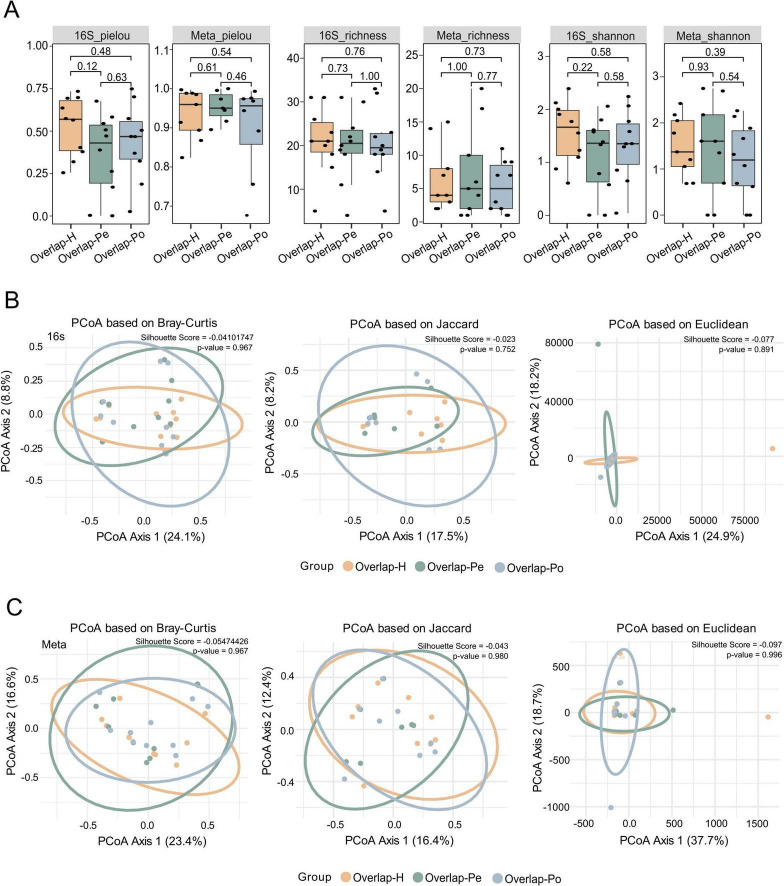
Identification of a compartment-independent core microbiota consistently detected by both sequencing platforms. **(A)** Alpha diversity indices of overlapping bacterial genera detected by both 16S rRNA sequencing (left) and shotgun metagenomics (right), including Pielou’s evenness, richness, and Shannon diversity across groups H, Pe, and Po. Statistical significance was evaluated using Wilcoxon rank-sum tests, with *p*-values annotated. **(B)** Principal coordinate analysis (PCoA) of beta diversity for overlapping genera identified in the 16S sequencing data, based on Bray-Curtis, Jaccard, and Euclidean distance metrics. **(C)** PCoA of beta diversity for overlapping genera identified in shotgun metagenomic sequencing, using the same three distance metrics. Ellipses indicate 95% confidence intervals, with Silhouette scores and *p*-values annotated.

We next focused on a subset of genera consistently detected across two sequencing platforms, which defined a stable “core microbiota” (listed in Supplementary Table 4). Diversity metrics calculated from this shared core remained stable across compartments, with no significant differences observed in alpha diversity (Figure 4A) (*P* > 0.05) or beta diversity (Figures 4B, C; Supplementary Figure 10) (*P* > 0.05). Despite methodological differences, these results collectively support the existence of a compartment-independent core microbiome in circulating blood.

## 4 Discussion

This study presents the first systematic comparison of 16S rRNA and shotgun metagenomic sequencing for the circulating microbiome. In 30 blood samples of our study, metagenomics (at ∼10 times of human genome coverage, 7,024,580,376 raw reads in total) identified just 76 genera, while 16S rRNA sequencing (6,612,678 raw reads in total) identified 240 genera from the same samples. Further increasing sequencing depth and sample size may enhance the performance of metagenomic sequencing for the circulating microbiome. This is exemplified by the work of Tan et al., who identified 1,818 genera from 9,770 samples sequenced to a depth of 15–30 times of human genome coverage (10). Such deep sequencing and large sample size is necessary because blood samples are characterized by low microbial biomass and a high host-to-microbial DNA ratio. Nevertheless, this high-resource strategy is frequently impractical without enough sample numbers or budgets.

Compared with the findings of Bars-Cortina et al. and Durazzi et al. in fecal samples, our observations in blood samples highlight the context-dependent differences between 16S rRNA and shotgun metagenomic sequencing (29, 30). In fecal samples with high microbial load and low host DNA contamination, shotgun sequencing identified over 500 genera, whereas 16S sequencing detected around 200 genera from the same specimens (29, 30). However, in our study, shotgun sequencing of low-biomass blood samples yielded limited taxonomic recovery, identifying only 76 genera after host-DNA removal, while 16S sequencing revealed 240 genera from the same cohort. This discrepancy highlights the critical influence of sample type: stool, with its high microbial biomass and limited host DNA contamination, is well suited to shotgun sequencing, whereas blood, low in microbial DNA and dominated by host DNA, is more effectively profiled by 16S amplicon sequencing (30, 31).

These divergent findings emphasize that 16S and shotgun metagenomics cannot be regarded as interchangeable tools, and highlight the need for methodological selection to be context-dependent. Shotgun sequencing tends to offer improved taxonomic and functional profiling when applied to microbiomes with high microbial load. However, for low-biomass samples, such as blood, 16S rRNA sequencing remains the more practical approach for microbial detection. This perspective is consistent with the findings of Marascio et al., who emphasized the clinical importance of early microbiome profiling in critical illness such as sepsis. Their study demonstrated that 16S-based methods can reveal clinically relevant microbial signals even in complex, DNA-poor specimens (32).

To clarify the causes of the discrepancies between the two sequencing methods, we examined the genera detected only by shotgun sequencing. Two potential sources within the 16S pipeline were considered: primer bias and chimeric artifacts. Primer bias, caused by mismatches between primers and target regions, can entirely prevent amplification of specific taxa (33). Chimeric sequences are artificial recombinants formed when fragments from different templates are erroneously joined during PCR, and subsequent chimera removal can occasionally discard true biological reads, particularly for low-abundance taxa (34, 35).

The *in silico* primer-bindng analysis confirmed that all shotgun-only taxa lacked complementarity to the 338F/806R primer pair, providing a direct mechanistic explanation for their absence from the 16S dataset. Chimeric artifacts are therefore unlikely to be responsible, as these taxa were not amplified in the first place.

Moreover, the shotgun-exclusive genera were sporadic and typically confined to a small subset of samples, consistent with the notion that these taxa represent rare or transient microbial signals rather than stable community members. Taken together, these findings indicate that primer mismatches and the stochastic occurrence of low-abundance taxa may account for the discrepancies observed between 16S and shotgun metagenomic sequencing.

As with balanced sensitivity, cost-efficiency, and technical feasibility, 16S rRNA sequencing has become the preferred method for blood microbiome profiling in conditions such as end-stage renal disease, hepatocellular carcinoma, and autoimmune disorders (1, 4, 5, 36–39). Our findings further indicates that 16S rRNA sequencing remains the most viable approach for circulating microbiome studies. However, there are still huge challenges. Circulating microbiome is prone to contamination originating from laboratory reagents, extraction kits, or environmental exposures (6, 31, 40–43). While negative controls were implemented to mitigate contamination, the possibility of reagent-derived artifacts remains. Moreover, both 16S rRNA and shotgun metagenomic sequencing rely exclusively on DNA-based detection, which precludes any direct assessment of microbial viability or metabolic activity (9, 13–15, 33, 44). The presence of microbial DNA alone does not distinguish between live bacteria and cell-free DNA fragments derived from microbial translocation or degradation (16, 33, 45).

Both sequencing methods revealed no significant alpha or beta diversity differences among portal, hepatic, and peripheral blood compartments, consistent with the findings of Païssé et al., Schierwagen et al. and Gedgaudas et al. (2, 3, 7). Moreover, we found the method-independent core microbiota in our study. These conserved core microbial communities also exhibited stable α- and β-diversity indices across separate vascular compartments. The consistent existence of a stable core microbiota across blood compartments suggests that the microbiome in peripheral blood is representative of the systemic microbiome, including the portal and hepatic regions. Therefore, we concluded that peripheral blood sampling may serve as a less invasive but reliable proxy for systemic microbiome profiling. Such insight is particularly relevant for clinical translation, where invasiveness and reproducibility are major constraints.

Next-generation approaches—such as culturomics, single-cell bacterial sequencing and molecular-sensing technologies—are crucial for accurately assessing microbial viability and metabolic function (46–49). Our findings emphasize the significant challenges inherent to characterizing the circulating microbiome. However, overcoming these barriers is essential not only for improving the detection of low-biomass signals, but also for advancing translational applications in the future.

## 5 Conclusion

In our study, 16S rRNA amplicon sequencing captured more diverse microbial signals than shotgun metagenomics. A stable microbial community structure was observed across vascular compartments, suggesting a homogeneous microbial composition throughout the circulatory system.

## Data Availability

The raw sequence data reported in this paper have been deposited in the Genome Sequence Archive (Genomics, Proteomics & Bioinformatics 2025) in National Genomics Data Center (Nucleic Acids Res 2025), China National Center for Bioinformation/Beijing Institute of Genomics, Chinese Academy of Sciences (GSA-Human: HRA014767) that are publicly accessible at https://ngdc.cncb.ac.cn/gsa-human (50, 51,).

## Footnotes

1
http://cctcc.whu.edu.cn/portal/dictionary/index

## References

[1] Lelouvier B Servant F Païssé S Brunet AC Benyahya S Serino M Changes in blood microbiota profiles associated with liver fibrosis in obese patients: a pilot analysis. *Hepatology*. (2016) 64:2015–27. 10.1002/hep.28829 27639192

[2] Gedgaudas R Bajaj JS Skieceviciene J Varkalaite G Jurkeviciute G Gelman S Circulating microbiome in patients with portal hypertension. *Gut Microbes*. (2022) 14:2029674. 10.1080/19490976.2022.2029674 35130114 PMC8824227

[3] Schierwagen R Alvarez-Silva C Madsen MSA Kolbe CC Meyer C Thomas D Circulating microbiome in blood of different circulatory compartments. *Gut*. (2019) 68:578–80. 10.1136/gutjnl-2018-316227 29581241

[4] Cho EJ Leem S Kim SA Yang J Lee YB Kim SS Circulating microbiota-based metagenomic signature for detection of hepatocellular carcinoma. *Sci Rep*. (2019) 9:7536. 10.1038/s41598-019-44012-w 31101866 PMC6525191

[5] Zhang Y Zhao R Shi D Sun S Ren H Zhao H Characterization of the circulating microbiome in acute-on-chronic liver failure associated with hepatitis B. *Liver Int*. (2019) 39:1207–16. 10.1111/liv.14097 30864226

[6] Hornung BVH Zwittink RD Ducarmon QR Kuijper EJ. Response to: ‘Circulating microbiome in blood of different circulatory compartments’ by Schierwagen et al. *Gut*. (2020) 69:789–90. 10.1136/gutjnl-2019-318601 30954950

[7] Païssé S Valle C Servant F Courtney M Burcelin R Amar J Comprehensive description of blood microbiome from healthy donors assessed by 16S targeted metagenomic sequencing. *Transfusion*. (2016) 56:1138–47. 10.1111/trf.13477 26865079

[8] Molina NM Sola-Leyva A Haahr T Aghajanova L Laudanski P Castilla JA Analysing endometrial microbiome: methodological considerations and recommendations for good practice. *Hum Reprod*. (2021) 36:859–79. 10.1093/humrep/deab009 33532852

[9] Gu W Miller S Chiu CY. Clinical metagenomic next-generation sequencing for pathogen detection. *Annu Rev Pathol Mech Dis*. (2019) 14:319–38. 10.1146/annurev-pathmechdis-012418-012751 30355154 PMC6345613

[10] Tan CCS Ko KKK Chen H Liu J Loh M. SG10K_Health Consortium, No evidence for a common blood microbiome based on a population study of 9,770 healthy humans. *Nat Microbiol*. (2023) 8:973–85. 10.1038/s41564-023-01350-w 36997797 PMC10159858

[11] Abellan-Schneyder I Matchado MS Reitmeier S Sommer A Sewald Z Baumbach J Primer, pipelines, parameters: issues in 16S rRNA gene sequencing. *mSphere*. (2021) 6:e1202–20. 10.1128/mSphere.01202-20 33627512 PMC8544895

[12] López-Aladid R Fernández-Barat L Alcaraz-Serrano V Bueno-Freire L Vázquez N Pastor-Ibáñez R Determining the most accurate 16S rRNA hypervariable region for taxonomic identification from respiratory samples. *Sci Rep*. (2023) 13:3974. 10.1038/s41598-023-30764-z 36894603 PMC9998635

[13] Regueira-Iglesias A Balsa-Castro C Blanco-Pintos T Tomás I. Critical review of 16S rRNA gene sequencing workflow in microbiome studies: From primer selection to advanced data analysis. *Mol Oral Microbiol*. (2023) 38:347–99. 10.1111/omi.12434 37804481

[14] Chiu CY Miller SA. Clinical metagenomics. *Nat Rev Genet*. (2019) 20:341–55. 10.1038/s41576-019-0113-7 30918369 PMC6858796

[15] Liu Y Ma Y. Clinical applications of metagenomics next-generation sequencing in infectious diseases. *J Zhejiang Univ Sci B*. (2024) 25:471–84. 10.1631/jzus.B2300029 38910493 PMC11199093

[16] Quince C Walker AW Simpson JT Loman NJ Segata N. Shotgun metagenomics, from sampling to analysis. *Nat Biotechnol*. (2017) 35:833–44. 10.1038/nbt.3935 28898207

[17] Rössle M Haag K Ochs A Sellinger M Nöldge G Perarnau JM The transjugular intrahepatic portosystemic stent-shunt procedure for variceal bleeding. *N Engl J Med*. (1994) 330:165–71. 10.1056/NEJM199401203300303 8264738

[18] Mortensen C Karlsen S Grønbæk H Nielsen DT Frevert S Clemmesen JO No difference in portal and hepatic venous bacterial DNA in patients with cirrhosis undergoing transjugular intrahepatic portosystemic shunt insertion. *Liver Int*. (2013) 33:1309–15. 10.1111/liv.12205 23763259

[19] Dantas Machado AC Ramos SF Gauglitz JM Fassler AM Petras D Aksenov AA Portosystemic shunt placement reveals blood signatures for the development of hepatic encephalopathy through mass spectrometry. *Nat Commun*. (2023) 14:5303. 10.1038/s41467-023-40741-9 37652904 PMC10471626

[20] Arab JP Martin-Mateos RM Shah VH. Gut-liver axis, cirrhosis and portal hypertension: the chicken and the egg. *Hepatol Int*. (2018) 12(Suppl 1):24–33. 10.1007/s12072-017-9798-x 28550391 PMC6876989

[21] Chen S. Ultrafast one-pass FASTQ data preprocessing, quality control, and deduplication using fastp. *iMeta.* (2023) 2:e107. 10.1002/imt2.107 38868435 PMC10989850

[22] Li H Durbin R. Fast and accurate short read alignment with Burrows-Wheeler transform. *Bioinformatics*. (2009) 25:1754–60. 10.1093/bioinformatics/btp324 19451168 PMC2705234

[23] Wood DE Lu J Langmead B. Improved metagenomic analysis with Kraken 2. *Genome Biol*. (2019) 20:257. 10.1186/s13059-019-1891-0 31779668 PMC6883579

[24] Lu J Breitwieser FP Thielen P Salzberg SL. Bracken: estimating species abundance in metagenomics data. *PeerJ Comput Sci*. (2017) 3:e104. 10.7717/peerj-cs.104 40271438 PMC12016282

[25] Rognes T Flouri T Nichols B Quince C Mahé F. VSEARCH: a versatile open source tool for metagenomics. *PeerJ*. (2016) 4:e2584. 10.7717/peerj.2584 27781170 PMC5075697

[26] Edgar RC. Search and clustering orders of magnitude faster than BLAST. *Bioinformatics*. (2010) 26:2460–1. 10.1093/bioinformatics/btq461 20709691

[27] Martin M. Cutadapt removes adapter sequences from high-throughput sequencing reads. *EMBnet j*. (2011) 17:10. 10.14806/ej.17.1.200

[28] Shen W Le S Li Y Hu F. SeqKit: a cross-platform and ultrafast toolkit for FASTA/Q file manipulation. Zou Q, ed. *PLoS One*. (2016) 11:e0163962. 10.1371/journal.pone.0163962 27706213 PMC5051824

[29] Bars-Cortina D Ramon E Rius-Sansalvador B Guinó E Garcia-Serrano A Mach N Comparison between 16S rRNA and shotgun sequencing in colorectal cancer, advanced colorectal lesions, and healthy human gut microbiota. *BMC Genomics*. (2024) 25:730. 10.1186/s12864-024-10621-7 39075388 PMC11285316

[30] Durazzi F Sala C Castellani G Manfreda G Remondini D De Cesare A. Comparison between 16S rRNA and shotgun sequencing data for the taxonomic characterization of the gut microbiota. *Sci Rep*. (2021) 11:3030. 10.1038/s41598-021-82726-y 33542369 PMC7862389

[31] Eisenhofer R Minich JJ Marotz C Cooper A Knight R Weyrich LS. Contamination in low microbial biomass microbiome studies: issues and recommendations. *Trends Microbiol*. (2019) 27:105–17. 10.1016/j.tim.2018.11.003 30497919

[32] Marascio N Scarlata GGM Romeo F Cicino C Trecarichi EM Quirino A The role of gut microbiota in the clinical outcome of septic patients: state of the art and future perspectives. *IJMS*. (2023) 24:9307. 10.3390/ijms24119307 37298258 PMC10252956

[33] Johnson JS Spakowicz DJ Hong BY Petersen LM Demkowicz P Chen L Evaluation of 16S rRNA gene sequencing for species and strain-level microbiome analysis. *Nat Commun*. (2019) 10:5029. 10.1038/s41467-019-13036-1 31695033 PMC6834636

[34] Haas BJ Gevers D Earl AM Feldgarden M Ward DV Giannoukos G Chimeric 16S rRNA sequence formation and detection in Sanger and 454-pyrosequenced PCR amplicons. *Genome Res*. (2011) 21:494–504. 10.1101/gr.112730.110 21212162 PMC3044863

[35] Maidak BL Olsen GJ Larsen N Overbeek R McCaughey MJ Woese CR. The ribosomal database project (RDP). *Nucleic Acids Res*. (1996) 24:82–5. 10.1093/nar/24.1.82 8594608 PMC145599

[36] Sumida K Pierre JF Han Z Mims TS Potukuchi PK Yuzefpolskaya M Circulating microbial signatures and cardiovascular death in patients with ESRD. *Kidney Int Rep*. (2021) 6:2617–28. 10.1016/j.ekir.2021.07.023 34622101 PMC8484116

[37] Shah NB Allegretti AS Nigwekar SU Kalim S Zhao S Lelouvier B Blood microbiome profile in CKD: a pilot study. *CJASN*. (2019) 14:692–701. 10.2215/CJN.12161018 30962186 PMC6500932

[38] Puri P Liangpunsakul S Christensen JE Shah VH Kamath PS Gores GJ The circulating microbiome signature and inferred functional metagenomics in alcoholic hepatitis. *Hepatology*. (2018) 67:1284–302. 10.1002/hep.29623 29083504 PMC5867221

[39] Hammad DBM Hider SL Liyanapathirana VC Tonge DP. Molecular characterization of circulating microbiome signatures in rheumatoid arthritis. *Front Cell Infect Microbiol*. (2020) 9:440. 10.3389/fcimb.2019.00440 32039040 PMC6987042

[40] Kim SJ Moon JY Ahn JH Weon HY Hong SB Seok SJ Diaphorobacter aerolatus sp. nov., isolated from air, and emended description of the genus Diaphorobacter. *Int J Syst Evol Microbiol*. (2014) 64:513–7. 10.1099/ijs.0.051060-0 24105945

[41] Glassing A Dowd SE Galandiuk S Davis B Chiodini RJ. Inherent bacterial DNA contamination of extraction and sequencing reagents may affect interpretation of microbiota in low bacterial biomass samples. *Gut Pathog*. (2016) 8:24. 10.1186/s13099-016-0103-7 27239228 PMC4882852

[42] Laurence M Hatzis C Brash DE. Common contaminants in next-generation sequencing that hinder discovery of low-abundance microbes. *PLoS One*. (2014) 9:e97876. 10.1371/journal.pone.0097876 24837716 PMC4023998

[43] Liu Y Elworth RAL Jochum MD Aagaard KM Treangen TJ. De novo identification of microbial contaminants in low microbial biomass microbiomes with Squeegee. *Nat Commun*. (2022) 13:6799. 10.1038/s41467-022-34409-z 36357382 PMC9649624

[44] Laudadio I Fulci V Palone F Stronati L Cucchiara S Carissimi C. Quantitative assessment of shotgun metagenomics and 16S rDNA amplicon sequencing in the study of human gut microbiome. *OMICS*. (2018) 22:248–54. 10.1089/omi.2018.0013 29652573

[45] Knight R Vrbanac A Taylor BC Aksenov A Callewaert C Debelius J Best practices for analysing microbiomes. *Nat Rev Microbiol*. (2018) 16:410–22. 10.1038/s41579-018-0029-9 29795328

[46] Sanabria A Hjerde E Johannessen M Sollid JE Simonsen GS Hanssen AM. Shotgun-Metagenomics on positive blood culture bottles inoculated with prosthetic joint tissue: a proof of concept study. *Front Microbiol*. (2020) 11:1687. 10.3389/fmicb.2020.01687 32765476 PMC7380264

[47] Lewis WH Tahon G Geesink P Sousa DZ Ettema TJG. Innovations to culturing the uncultured microbial majority. *Nat Rev Microbiol*. (2021) 19:225–40. 10.1038/s41579-020-00458-8 33093661

[48] Lloréns-Rico V Simcock JA Huys GRB Raes J. Single-cell approaches in human microbiome research. *Cell*. (2022) 185:2725–38. 10.1016/j.cell.2022.06.040 35868276

[49] Zhang Q Hutchison ER Pan C Warren MF Keller MP Attie AD Systems genetics uncovers associations among host amylase locus, gut microbiome, and metabolic traits in mice. *Microbiome*. (2025) 13:101. 10.1186/s40168-025-02093-y 40259344 PMC12012960

[50] Zhang S Chen X Jin E Wang A Chen T Zhang X The GSA family in 2025: a broadened sharing platform for multi-omics and multimodal data. *Genom Proteom Bioinform*. (2025) 23:qzaf072. 10.1093/gpbjnl/qzaf072 40857552 PMC12451262

[51] CNCB-NGDC Members and Partners. Database resources of the National Genomics Data Center, China National Center for Bioinformation in 2025. *Nucleic Acids Res*. (2025) 53:D30–44. 10.1093/nar/gkae978 39530327 PMC11701749

