# Supplementary material for: Circulating microbiome profiling in transjugular intrahepatic portosystemic shunt patients: 16S rRNA vs. shotgun sequencing
Source: Front Med (Lausanne). 2025 Dec 4;12:1662837. doi: 10.3389/fmed.2025.1662837 (PMC12711707; doi:10.3389/fmed.2025.1662837)
Supplement: Supplementary file 2 [file Image_1.pdf]

Supplementary Material

Supplementary Figures

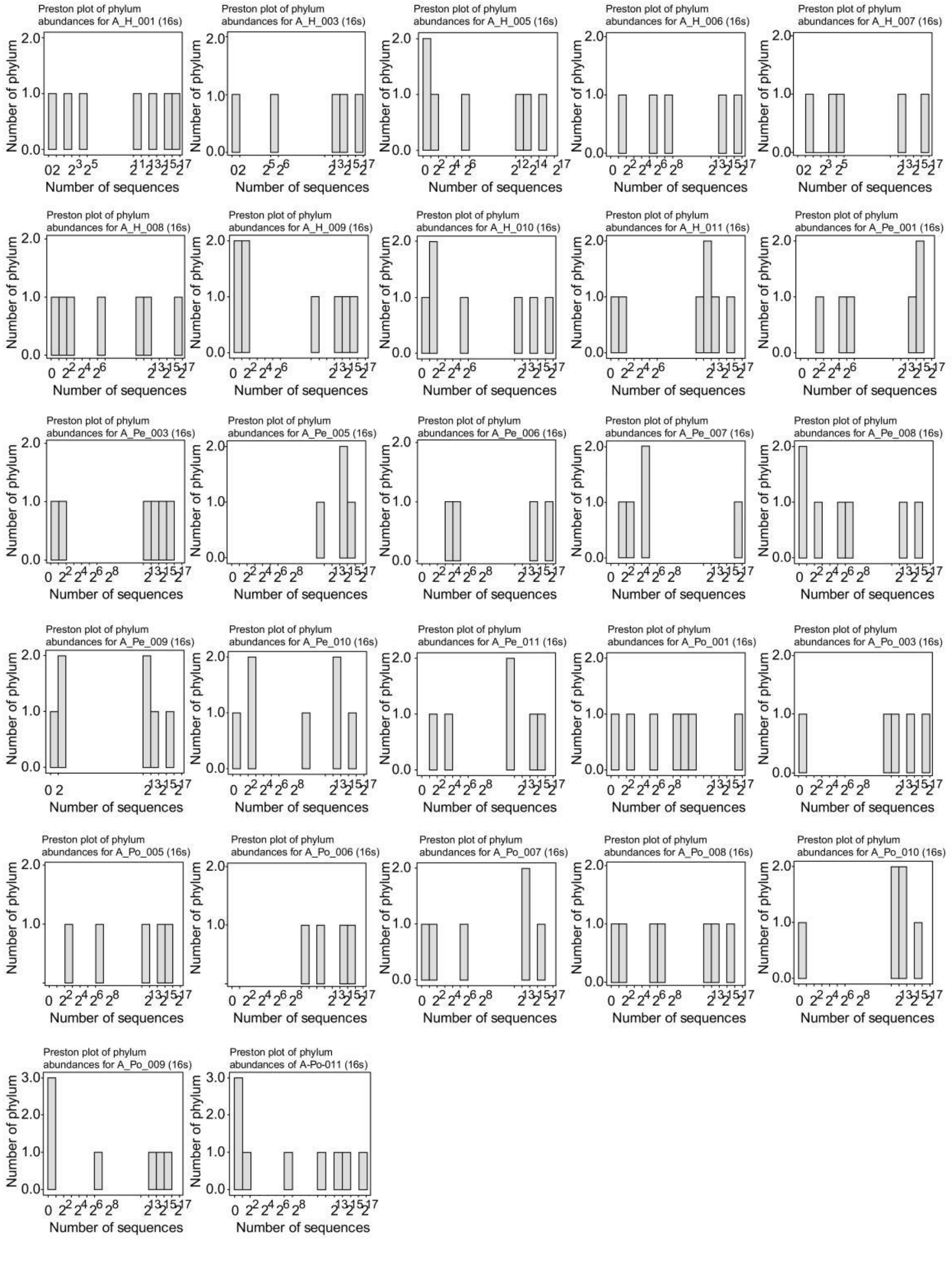

Fig. S1 Preston plot of phylum abundances in all 16s samples from different blood compartment. Each panel represents a single sample, labeled by patient ID, with the x-axis indicating the $\log_2$-binned number of sequences per phylum and the y-axis representing the number of phyla observed in each panel.

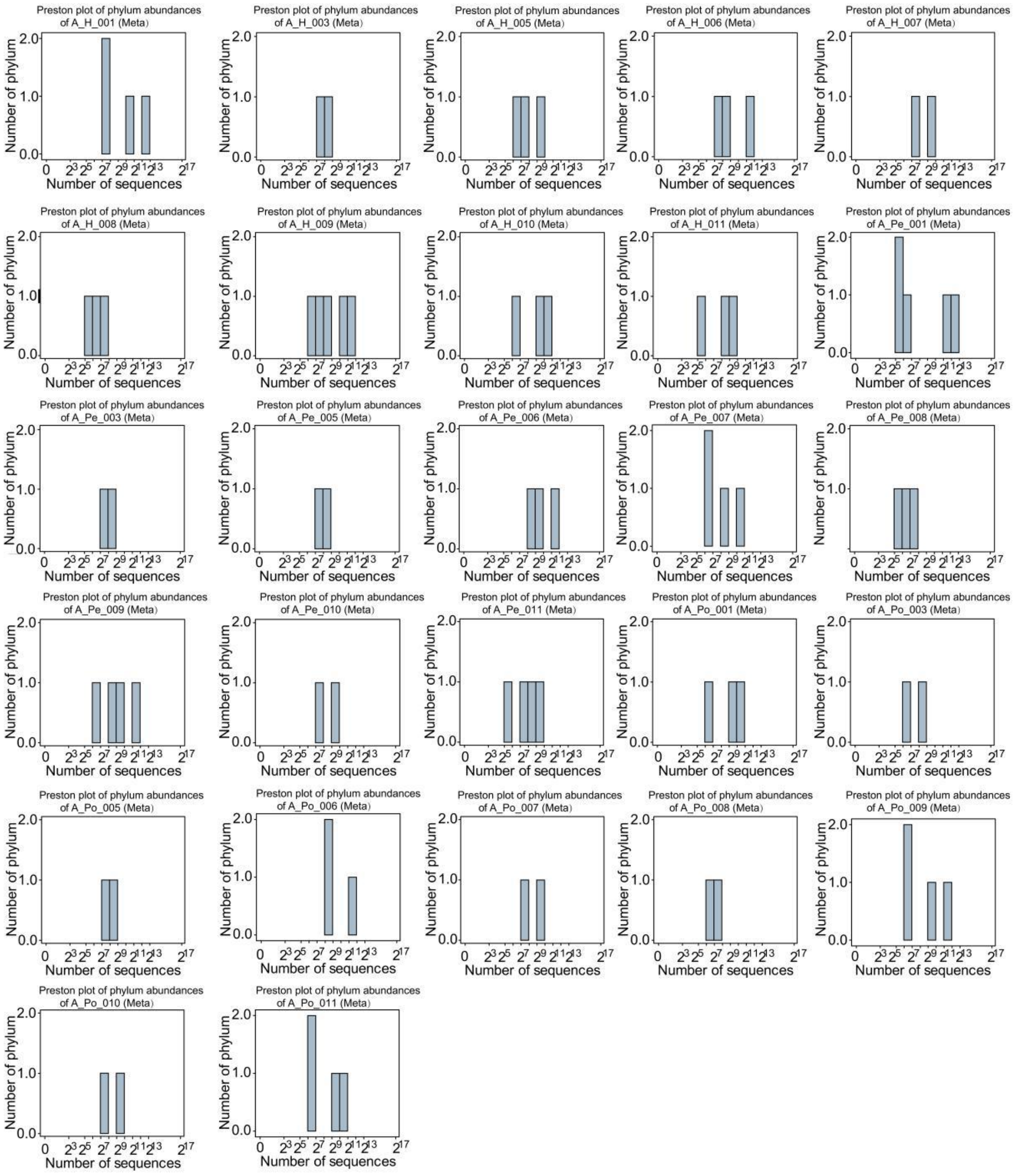

Fig. S2 Preston plots of phylum-level abundances in shotgun metagenomic datasets from different blood compartments.

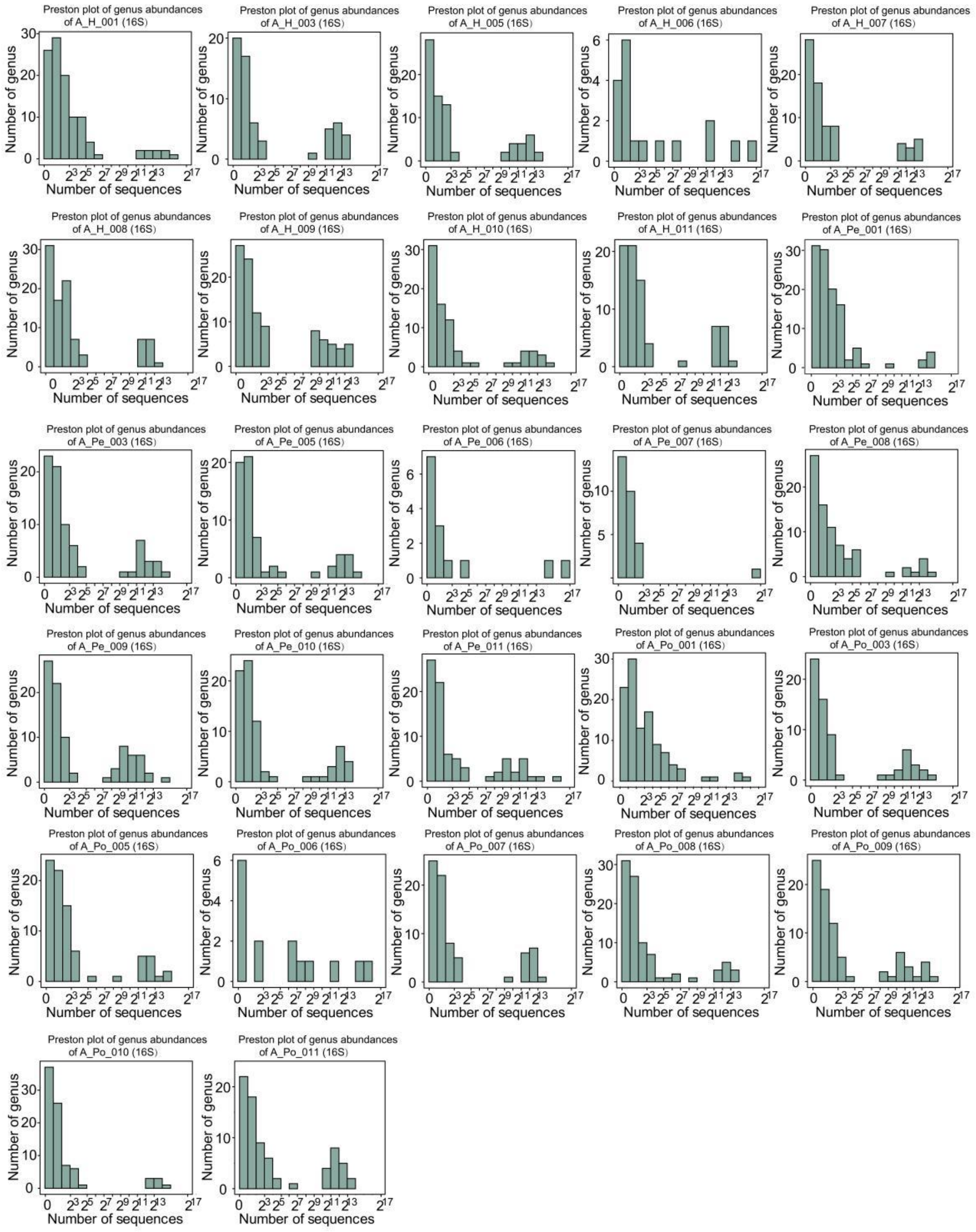

Fig. S3 Preston plots of genus-level abundances in 16s rRNA sequencing datasets from different blood compartments.

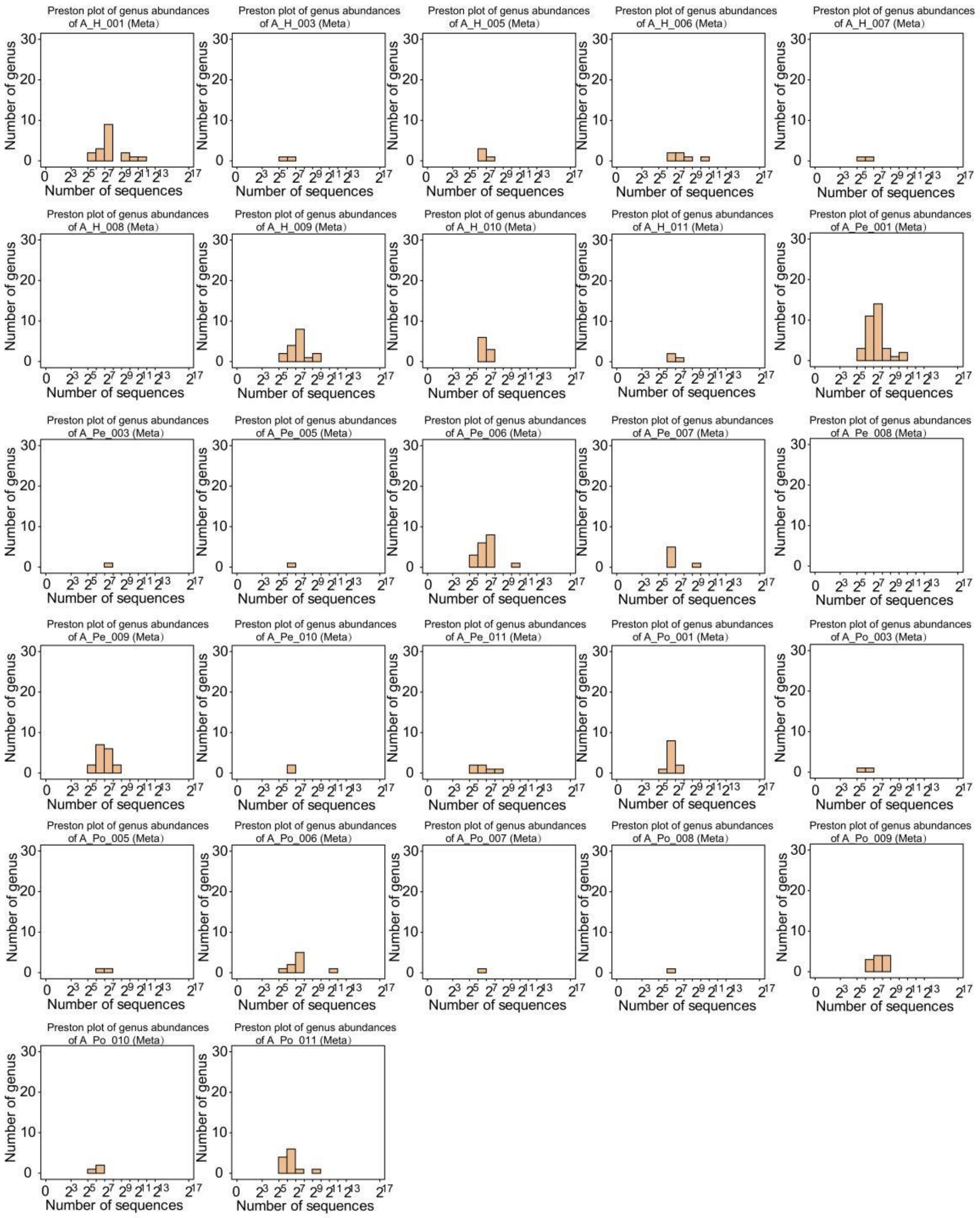

Fig. S4 Preston plots of genus-level abundances in shotgun metagenomic datasets from different blood compartments.

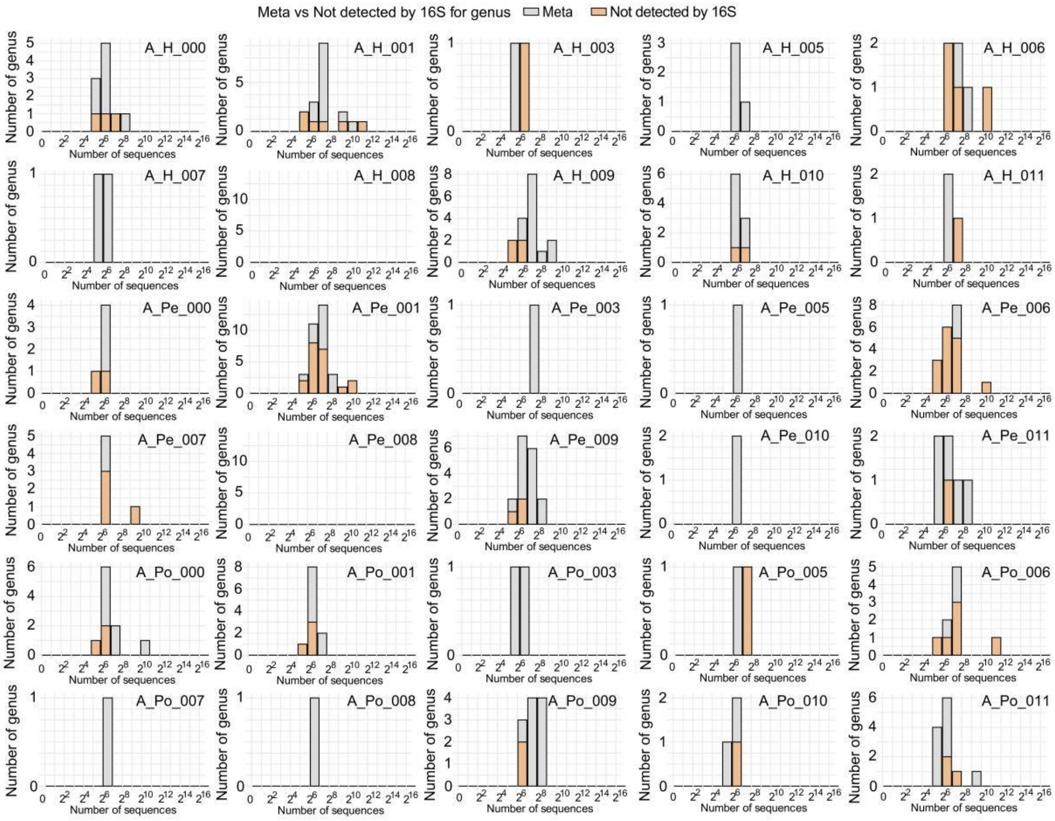

Fig. S5 Comparison of Genera Detected by Shotgun Metagenomic Sequencing and Not Detected by 16S rRNA Sequencing. Histograms display the number of genera detected by shotgun metagenomic sequencing (gray) and those not detected by 16S rRNA sequencing (orange) across various samples. The x-axis represents the number of sequences on a log2 scale, and the y-axis indicates the number of genera. This comparison highlights the genera identified by shotgun metagenomics but missed by 16S rRNA sequencing, reflecting differences in detection sensitivity between the two methods.

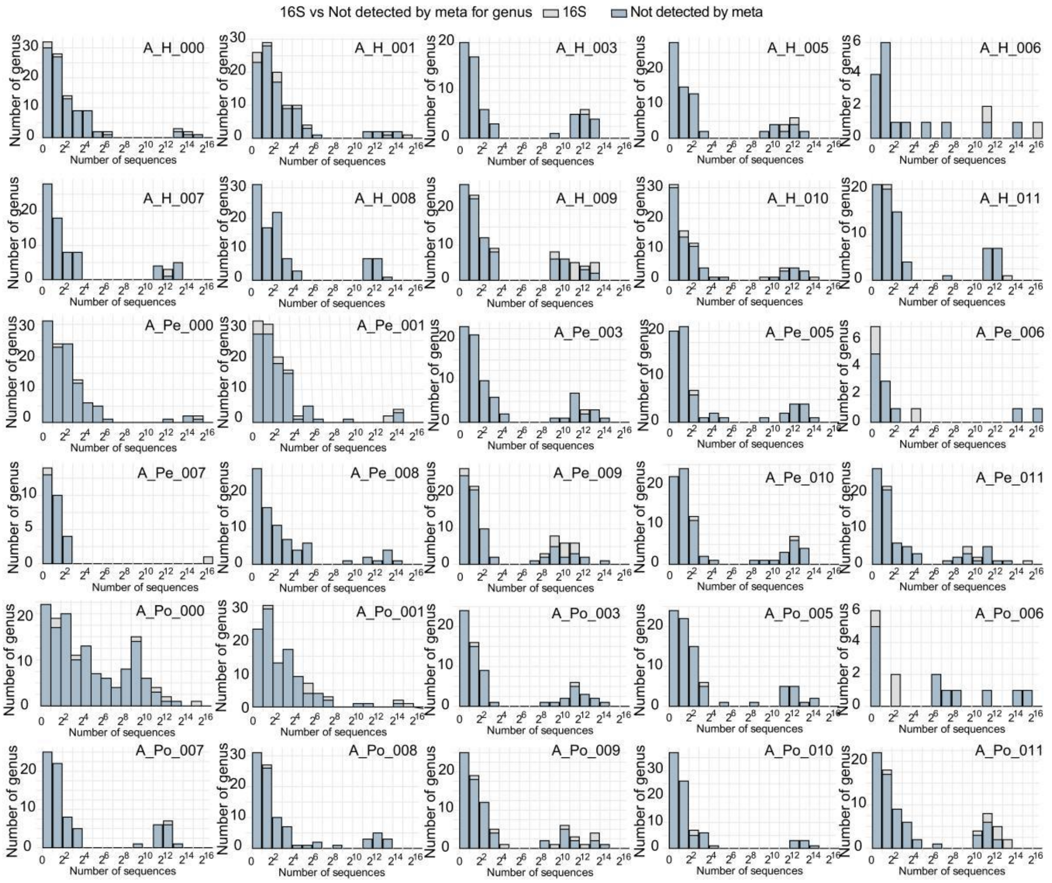

Fig. S6 Comparison of Genera Detected by 16S rRNA Sequencing and Not Detected by Shotgun Metagenomic Sequencing. Histograms show the number of genera detected by 16S rRNA sequencing (gray) and those not detected by shotgun metagenomic sequencing (blue) across the same set of samples. The x-axis presents the number of sequences (log2 scale), and the y-axis shows the number of genera detected. This figure emphasizes the genera identified by 16S rRNA sequencing but absent in shotgun metagenomic results, indicating possible biases in detection due to methodological differences.

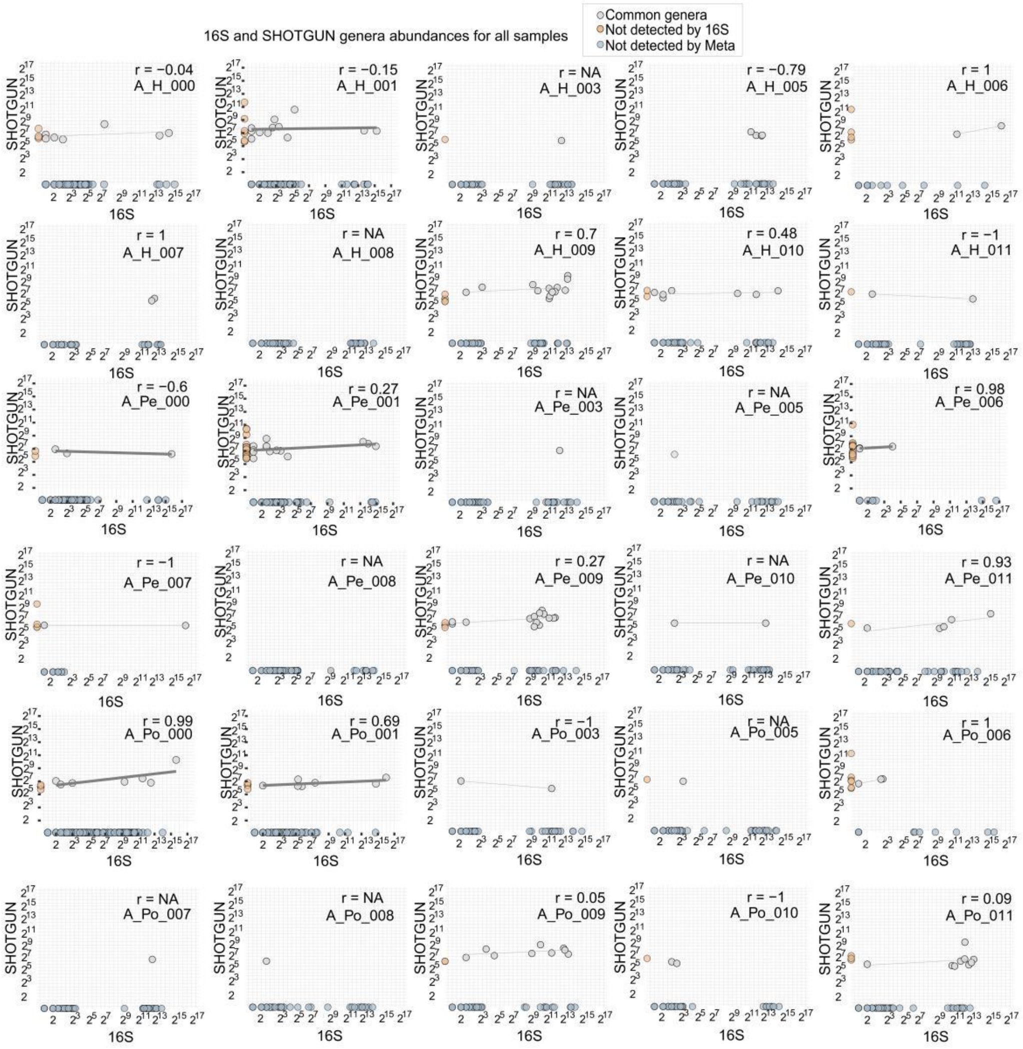

Fig. S7 Overall Comparison of Genus Detection Between Shotgun Metagenomic Sequencing and 16S rRNA Sequencing. A comprehensive comparison of genus detection between shotgun metagenomic sequencing and 16S rRNA sequencing across multiple samples.

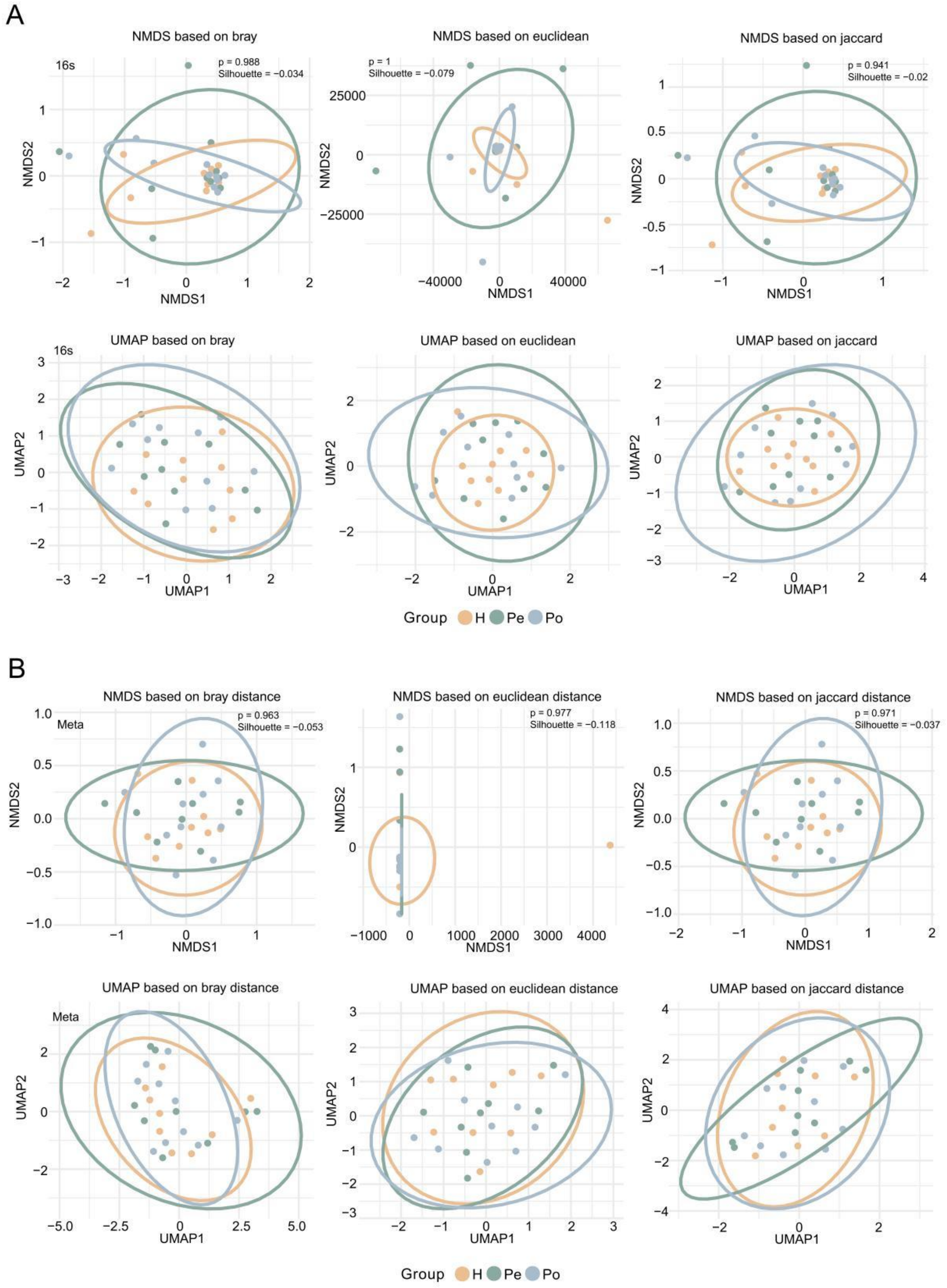

Fig. S8 (A) Non-metric multidimensional scaling (NMDS) and uniform manifold approximation and projection (UMAP) ordinations of beta diversity for 16S rRNA sequencing data, based on Bray-Curtis, Euclidean, and Jaccard distance metrics, comparing microbial communities among hepatic (H), peripheral (Pe), and portal (Po) vein samples. **(B)** NMDS and UMAP ordinations of beta diversity for shotgun metagenomic sequencing data using the same three distance metrics, similarly assessing differences among groups. Ellipses indicate 95% confidence intervals, with Silhouette scores provided as measures of clustering.

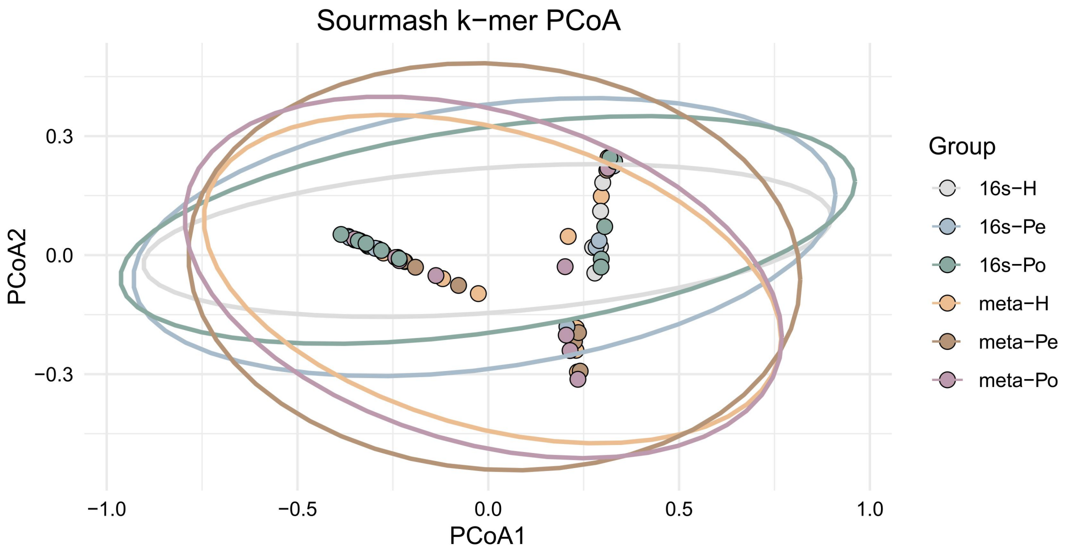

Fig. S9 Principal coordinate analysis (PCoA) based on k-mer similarity profiles computed with *Sourmash*, comparing microbial community structures across hepatic (H), peripheral (Pe), and portal (Po) vein samples using both 16S rRNA amplicon sequencing and shotgun metagenomics. Ellipses represent 95% confidence intervals for each group.

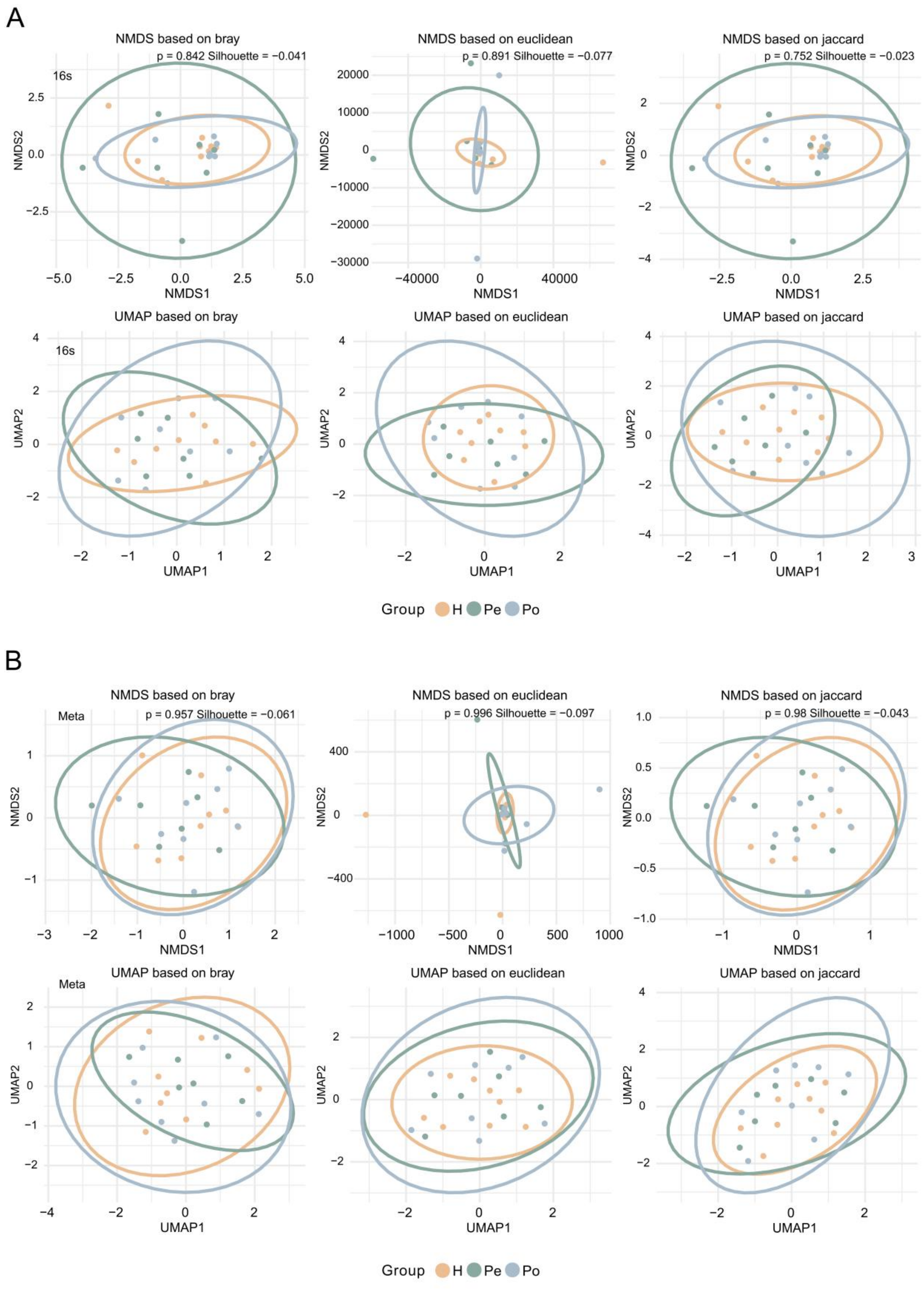

Fig. S10 (A) Non-metric multidimensional scaling (NMDS) and uniform manifold approximation and projection (UMAP) ordinations of beta diversity for overlapping genera identified in the 16S rRNA sequencing data, based on Bray – Curtis, Euclidean, and Jaccard distance metrics. **(B)** NMDS and UMAP ordinations of beta diversity for overlapping genera identified in shotgun metagenomic sequencing, using the same

three distance metrics. Ellipses represent 95% confidence intervals, with Silhouette scores and p-values annotated.
